# Multimodal imaging techniques in Yttrium-90 radioembolization for hepatocellular carcinoma: a modality-oriented clinical review

**DOI:** 10.3389/fonc.2026.1748836

**Published:** 2026-02-03

**Authors:** Tingting Yang, Lijie Zhang, Wei Xu, Chuansheng Zheng, Bin Liang

**Affiliations:** 1 Department of Radiology, Union Hospital, Tongji Medical College, Huazhong University of Science and Technology, Wuhan, China; 2 Hubei Provincial Clinical Research Center for Precision Radiology & Interventional Medicine, Wuhan, China; 3 Hubei Province Key Laboratory of Molecular Imaging, Wuhan, China

**Keywords:** 90Y, clinical application, hepatocellular carcinoma, multimodal imaging, radioembolization

## Abstract

Hepatocellular carcinoma (HCC) is one of the most common malignancies with high global mortality. Yttrium-90 selective internal radiation therapy (^90^Y-SIRT) is a precision radio-interventional treatment whose efficacy and safety critically depend on accurate microsphere delivery and dose distribution. This review provides an overview of imaging techniques used in ^90^Y-SIRT, emphasizing both single-modality and multimodal approaches. We summarize the clinical value of ultrasound, CT, MRI, angiography, and nuclear medicine, each providing anatomical, functional, or metabolic information. We further discuss multimodal imaging integration across the treatment workflow: pre-procedural fusion supports patient selection and dose planning; intraprocedural volumetric imaging guides catheter placement; and post-treatment functional imaging assesses microsphere distribution and therapeutic response. By consolidating current evidence, this review highlights the clinical utility of multimodal imaging and identifies areas for optimization in treatment planning, procedural guidance, and outcome assessment of ^90^Y-SIRT. This synthesis serves as a practical reference for clinicians and researchers aiming to improve the precision and effectiveness of radioembolization for HCC.

## 1 Introduction

Hepatocellular carcinoma (HCC) is the sixth most common malignancy and the third leading cause of cancer-related deaths worldwide (1). Due to its insidious clinical presentation, a substantial proportion of patients are diagnosed at intermediate or advanced stages, and less than 30% are candidates for curative treatments at initial diagnosis (2, 3). Consequently, locoregional therapies play a pivotal role in the multidisciplinary management of HCC (4). Yttrium-90 selective internal radiation therapy (^90^Y-SIRT) has emerged as an important locoregional treatment option that can be applied across various stages of HCC (5, 6). By selectively delivering radioactive microspheres into tumor-feeding arteries, ⁠^90^Y-SIRT achieves high-dose internal irradiation of tumor tissue through the emission of high-energy β-particles, while relatively sparing non-tumorous liver parenchyma (7). Nevertheless, given the high radioactivity of ^90^Y microspheres, treatment efficacy and safety are critically dependent on precise microsphere delivery (8). Inaccurate administration or ectopic embolization may lead to severe complications or even death (8, 9). As a result, the successful implementation of ⁠^90^Y-SIRT requires meticulous patient selection, accurate pre-procedural assessment of vascular anatomy, microsphere distribution, and dosimetry, as well as reliable intra-procedural guidance and post-treatment monitoring (5). In clinical practice, however, individual imaging modalities each provide complementary but incomplete information regarding tumor anatomy, vascular complexity, extrahepatic shunting, and microsphere distribution (10). Conventional anatomical imaging, angiographic techniques, and nuclear medicine imaging each provide distinct but inherently limited information, particularly with regard to accurately depicting microsphere distribution and predicting radiation dose delivery (10). Multimodal imaging, defined as the integration of two or more imaging modalities into a single framework, enables the fusion of anatomical, functional, and molecular information, providing more accurate diagnostic and therapeutic guidance than any single imaging technique (11). In the context of ^90^Y-SIRT, multimodal imaging supports the entire treatment continuum.

**Figure 1:**
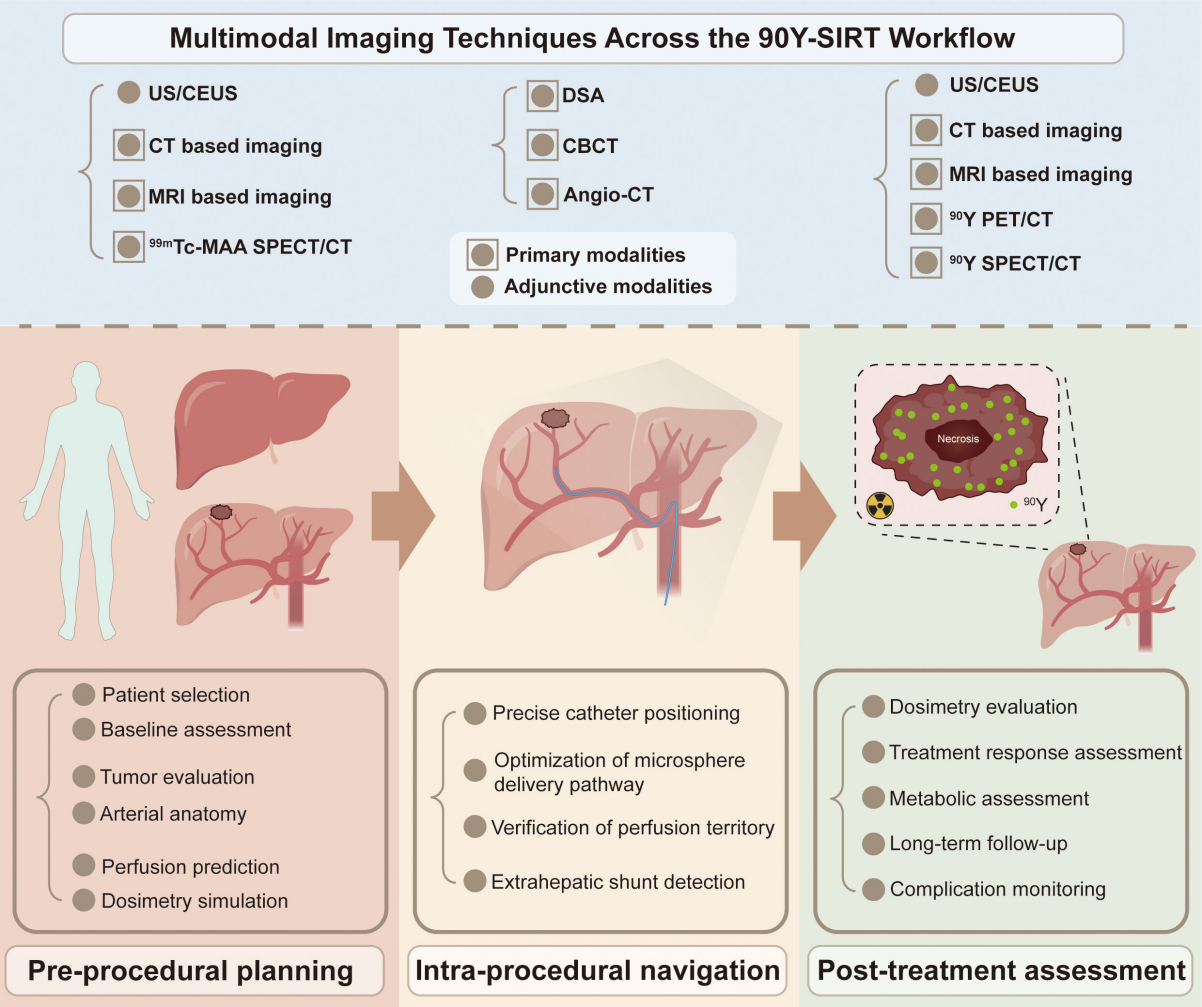
Multimodal imaging techniques across the yttrium-90 selective internal radiation therapy (^90^Y-SIRT) workflow for hepatocellular carcinoma. This schematic summarizes the roles of different imaging modalities at each stage of ^90^Y-SIRT, from pre-procedural planning through intra-procedural navigation to post-treatment assessment. Solid circles denote core imaging guiding therapy; circles with surrounding squares indicate complementary or optional imaging. Abbreviations: ^90^Y-SIRT, yttrium-90 selective internal radiotherapy; US, ultrasound; CEUS, contrast-enhanced ultrasound; CBCT, cone-beam CT; Angio-CT, angiography CT; PET/CT, positron emission tomography/CT, SPECT/CT, single-photon emission computed tomography/CT.

This review aims to provide a modality-oriented overview of imaging techniques used in ⁠^90^Y-SIRT for HCC, summarizing the clinical value of single-modality imaging and the role of multimodal imaging in procedural guidance and treatment optimization. By systematically integrating information across different imaging modalities, this review highlights their complementary strengths and provides a comprehensive perspective on how multimodal imaging can improve treatment planning, dose delivery, and outcome prediction. As shown in **Figure 1**, we outline recent advances and challenges in the application of multimodal imaging throughout the ^90^Y-SIRT workflow, highlighting its pivotal role in treatment planning, efficacy optimization, and the prospects for future development.

## 2 Cross-sectional anatomical imaging in ^90^Y-SIRT

### 2.1 US based imaging

Ultrasound (US) serves as the primary surveillance modality for patients at risk of HCC, acting as the initial screening tool before patients are considered for ^90^Y-SIRT. While B-mode US is non-invasive, its utility for treatment planning is restricted by variable image quality. Schoenberger et al. reported that nearly 18% of patients with cirrhosis present with moderate-to-severe visualization limitations, with high body mass index and non-alcoholic fatty liver disease identified as independent risk factors for inadequate imaging (12). Consequently, clinical guidelines mandate that any nodule ≥ 10 mm detected on US requires further characterization with multiphasic cross-sectional imaging to confirm the diagnosis (13, 14).

To address these limitations, contrast-enhanced ultrasound (CEUS) has been integrated into diagnostic algorithms to resolve indeterminate findings. Hawley et al. highlight that newer ultrasound contrast agents, such as perflubutane, possess a unique post-vascular Kupffer phase (15). These agents are phagocytosed by Kupffer cells in healthy parenchyma but not by malignant cells, enabling “defect reperfusion imaging” that facilitates whole-liver assessment and improves the detection of occult nodules missed by standard B-mode US (15).

In the specific workflow of ^90^Y-SIRT, CEUS provides a distinct advantage for early post-treatment monitoring. Unlike bland embolization, ^90^Y microspheres (20-30 μm) do not fully occlude tumor-feeding vessels, allowing residual intratumoral blood flow to be evaluated by CEUS (16). Delaney et al. demonstrated that quantitative CEUS could predict long-term response as early as 2 weeks post-treatment, significantly earlier than the standard 3–6 months interval required for morphological changes on CT or MRI (16). In their study, responders exhibited a significant decrease in fractional vascularity of approximately 38% at 2 weeks, whereas non-responders showed unchanged or increased vascularity (16). Thus, CEUS serves as a critical adjunctive modality for monitoring early hemodynamic changes following radioembolization.

### 2.2 CT based techniques

Multidetector computed tomography (MDCT) serves as the standard modality for the initial staging and vascular mapping of hepatocellular carcinoma (HCC) prior to Yttrium-90 (^90^Y) radioembolization. Due to its rapid acquisition speed and high spatial resolution, multiphasic CT is indispensable for defining hepatic arterial anatomy and calculating liver volumes for dosimetry. A recent multicenter study by Yoon et al. reported that contrast-enhanced CT achieved a sensitivity of approximately 75% for diagnosing HCC under LI-RADS criteria, performing comparably to gadoxetic acid-enhanced MRI in scenarios where washout timing is critical (17). However, conventional CT remains limited in characterizing small hypovascular nodules (< 2 cm) compared to MRI, and standard morphological evaluation often fails to predict microvascular invasionpreoperatively (18).

To enhance diagnostic capabilities, dual-energy CT (DECT) has been introduced to differentiate materials based on their energy-related attenuation characteristics. Silva et al. highlight that DECT can generate material density images, such as iodine and water maps (19). These maps allow for the reconstruction of virtual unenhanced images, potentially eliminating the need for a true non-contrast phase and reducing radiation dose (19). Furthermore, monochromatic images generated by DECT improve the contrast-to-noise ratio, aiding in the distinction of true contrast enhancement from hemorrhage or calcification, which is particularly valuable in complex post-treatment scenarios (19).

In the specific context of post-^90^Y-SIRT assessment, DECT addresses the limitations of size-based criteria, as radioembolization is cytostatic and tumors often do not shrink immediately despite necrosis. Altenbernd et al. demonstrated that quantifying iodine uptake via DECT is a superior biomarker for response compared to morphological criteria (20). In their prospective study, iodine uptake classification identified 26 partial responders, whereas standard American Association for the Study of Liver Diseases criteria identified only 8 responders within the same cohort (20). By visualizing the loss of vascularity rather than mere shrinkage, DECT provides a more accurate indication of therapeutic success (20).

Beyond spectral imaging, CT radiomics and CT liver perfusion (CTLP) offer functional insights into tumor microcirculation. Zhong et al. developed a radiomics model that achieved high accuracy (AUC 0.890) in preoperatively predicting microvascular invasion and relapse-free survival in HCC ≥ 3 cm (21). Regarding perfusion parameters, Hayano et al. showed that HCCs exhibit significantly higher blood volume than hypovascular metastases but lower values than hypervascular metastases (22). Additionally, Kalarakis et al. found that optimizing CTLP protocols could reduce radiation exposure by nearly 50% without compromising diagnostic accuracy (23). In post-treatment monitoring, Reiner et al. found that a significant decrease in arterial perfusion on CTLP 4 weeks after radioembolization correlated with improved 1-year survival in patients with liver metastases (24).

### 2.3 MRI based techniques

MRI serves as the reference standard for the diagnosis, treatment planning, and response assessment of HCC in the context of ^90^Y-SIRT, owing to its superior soft-tissue contrast and multiparametric functional capabilities. The application of hepatobiliary-specific contrast agents, such as gadoxetic acid (Gd-EOB-DTPA), significantly refines diagnostic accuracy by exploiting organic anion transporting polypeptides (OATP). Since HCC cells typically lack functional OATP1B3 expression, most HCC lesions appear hypointense during the hepatobiliary phase, thereby improving lesion conspicuity and facilitating the detection of iso- or hypovascular tumors (25). A head-to-head meta-analysis confirms this superiority, demonstrating a pooled sensitivity of 86% for Gd-EOB-DTPA-enhanced MRI compared to 70% for MDCT (26). This advantage is critical for small lesions; for tumors ≤ 1 cm, MRI sensitivity (46%) is more than double that of CT (20%) (26). Diagnostic performance of MRI remains guideline-dependent; applying Asian Pacific Association for the Study of the Liver criteria yields a sensitivity of 89.3%, whereas LI-RADS criteria reduce sensitivity to 70.6% but ensure a specificity of 90.4%, minimizing false-positive allocations for radioembolization (17).

Diffusion-weighted imaging (DWI) provides functional information by probing the random Brownian motion of water molecules, which is inversely correlated with tissue cellularity and cell membrane integrity. Malignant tumors typically exhibit restricted diffusion due to high cellular density, resulting in high signal intensity on high b-value images and low values on the quantitative apparent diffusion coefficient (ADC) map (25, 27). As an advanced application of diffusion sequences, intravoxel incoherent motion (IVIM) imaging utilizes multiple b-values to distinguish true molecular diffusion from perfusion-related pseudo-diffusion (28, 29). This allows for the simultaneous assessment of tissue cellularity and capillary microcirculation without the need for intravenous contrast, providing a unique non-invasive perspective on the tumor microenvironment prior to and following embolization (27).

Dynamic contrast-enhanced MRI (DCE-MRI) is the gold standard for the quantitative assessment of tumor vascularity, analyzing the pharmacokinetics of contrast agent distribution to derive parameters such as the volume transfer constant and arterial flow (29). Given that ^90^Y-SIRT relies on the exclusive arterial supply of HCCs, DCE-MRI provides direct mechanistic insights into therapeutic efficacy. Technological advancements like golden-angle radial sparse parallel (GRASP) acquisition have overcome traditional temporal resolution limitations (30). GRASP enables free-breathing perfusion imaging that yields quantitative parameters, such as arterial liver perfusion and hepatic perfusion index, which demonstrate excellent agreement with volume perfusion CT (ICC ≥ 0.88) but without the associated ionizing radiation (30).

For ^90^Y-SIRT procedural planning, precise target definition is a prerequisite for accurate dosimetry. Conventional anatomical sequences may suffer from inter-observer variability. The application of multisource adaptive MRI fusion, which computationally synthesizes T1-weighted, T2-weighted, and DWI data, elevates the tumor contrast-to-noise ratio to 9.82, vastly superior to the 0.72 of planning CT (31). This fusion technique improves the consistency of gross tumor volume delineation, achieving a Dice similarity coefficient of 0.95 (31, 32). Additionally, baseline texture analysis (radiomics) of DCE-MRI maps offers prognostic value; high intratumoral heterogeneity in arterial fraction has been identified as a robust predictor of long-term complete response to radioembolization (AUC = 0.857) [7].

Post-procedural response assessment with MRI addresses the inherent limitations of anatomical criteria. Following ^90^Y-SIRT, tumors often undergo coagulation necrosis without immediate shrinkage; studies indicate no significant change in tumor size at 30 days post-treatment (mean 9.4 cm to 9.2 cm), rendering standard RECIST criteria insensitive in the early phase [9]. Conversely, functional metrics respond rapidly. A reduction in arterial enhancement by approximately 22% is an early sign of devascularization [9]. Similarly, IVIM imaging can detect significant decreases in arterial flow and pseudodiffusion coefficients as early as 6 weeks post-treatment (28). Critically, quantitative ADC changes have prognostic value: an increase in ADC greater than 30% at 30 days post-treatment is a potent predictor of objective response (sensitivity 90%) and correlates with significantly prolonged median overall survival (13.9 months vs. 5.5 months in non-responders) (33). However, hemorrhagic necrosis can cause high T1 signal intensity on pre-contrast images, obscuring viable tumor enhancement. Image subtraction techniques effectively mitigate this confounder, improving the sensitivity for detecting complete tumor necrosis to 78.1%, compared to only 28.1% with non-subtracted images (34).

## 3 Angiographic imaging techniques in ^90^Y-SIRT

Angiographic imaging constitutes the procedural backbone of ^90^Y-SIRT, essential not only for intra-procedural guidance but also for pre-treatment vascular mapping and flow dynamic assessment. Once a patient is deemed eligible, a comprehensive angiographic evaluation is performed to document hepatic arterial anatomy and identify tumor-feeding vessels. This step is critical for detecting hazardous extrahepatic branches or anastomoses that could lead to non-target microsphere deposition, thereby allowing for catheter repositioning or prophylactic embolization when necessary (35).

### 3.1 Digital subtraction angiography

DSA remains the reference standard for pre-procedural vascular mapping. A thorough understanding of hepatic arterial variants is a prerequisite for successful radioembolization, as the standard anatomy (Michels Type I) accounts for only 55%-75% of cases (36, 37). Failure to recognize variants, such as replaced or accessory hepatic arteries, may compromise the accuracy of lung shunt estimation and result in incomplete tumor coverage. Furthermore, large peripherally located HCCs frequently recruit parasitic blood supply from extrahepatic sources, most commonly the right inferior phrenic artery (38). Overlooking these collateral feeders precludes complete tumor treatment and necessitates comprehensive angiographic interrogation beyond the celiac axis (38).

In the context of ^90^Y-SIRT, the primary utility of DSA lies in safety and hemodynamic assessment. It is essential for identifying vessels supplying the gastrointestinal tract, specifically the gastroduodenal artery, right gastric artery, and cystic artery—to prevent complications like gastric ulceration or cholecystitis (10, 38). Concurrently, DSA identifies tumor-feeding vessels and evaluates real-time hemodynamics to detect potential flow reversal caused by large hypervascular tumors; these findings collectively guide the optimal catheter positioning for the subsequent injection of Technetium-99m macroaggregated albumin (^99m^Tc-MAA) (38).

Despite its central role, DSA has inherent limitations. As a two-dimensional projection technique, it may obscure complex vascular overlaps and fail to detect small collateral vessels, necessitating the adjunctive use of volumetric imaging (10). Additionally, DSA is highly operator-dependent; its diagnostic accuracy and safety profile rely heavily on the interventionalist’s experience in recognizing subtle vascular anomalies and achieving stable microcatheter positioning for super-selective delivery.

### 3.2 Cone-beam CT and angiography CT

Volumetric intraprocedural imaging, encompassing CBCT and Angio-CT, has evolved from an adjunctive tool to a critical component of the ^90^Y-SIRT workflow. By providing three-dimensional soft-tissue visualization directly in the interventional suite, these modalities overcome the limitations of two-dimensional DSA in assessing vascular complexity and perfusion territories. The precise identification of tumor-feeding arteries and extrahepatic shunts is paramount for safety, yet reliance on DSA alone often results in the misclassification of vascular supply, particularly in anatomically complex regions like hepatic segments I and IV. In a study of 42 patients, Louie et al. found that CBCT revealed extrahepatic enhancement or incomplete tumor perfusion in 52% of cases, with 33% of these findings being exclusive to CBCT and undetectable by DSA (39). Heusner et al. further validated this diagnostic superiority, demonstrating that CBCT achieved a specificity of 96% and an accuracy of 90% for detecting extrahepatic shunts, compared to 88% and 81% for DSA, respectively (40). Consequently, intraprocedural volumetric imaging facilitates immediate preventative embolization of aberrant vessels, such as the falciform or right gastric arteries, significantly reducing the risk of gastrointestinal ulceration.

Beyond safety, volumetric imaging is essential for optimizing dosimetry by delineating the microcatheter-perfused liver volume, which often differs from anatomical lobes defined by venous landmarks on pre-procedural CT. Rhee et al. established that using catheter-directed CT angiography to calculate segmental volumes rather than standard lobar volumes resulted in a significantly higher calculated effective tumor dose (348 Gy vs. 100 Gy) (41). Grözinger et al. reported that angiographic prediction of segmental blood supply differed from the actual perfusion territory defined by volumetric imaging in 27.3% of patients, resulting in a mean mismatch volume of 90 ± 54 mL. This discrepancy led to dose calculation differences ranging from approximately 11% to 14% for both glass and resin microspheres, depending on the treated liver lobe (42). Furthermore, Jafargholi Rangraz et al. showed that discrepancies between anatomical and perfusion-based liver lobe segmentation derived from contrast-enhanced CBCT could result in substantial absorbed dose differences, reaching up to 21 Gy for the left lobe and 9 Gy for the right lobe (43). These findings highlight the importance of CBCT-based perfusion territory segmentation for accurate definition of liver vascular supply in dosimetric calculations.

While CBCT is widely available, it is limited by a restricted field of view, susceptibility to motion artifacts, and low contrast resolution. Angio-CT systems, which integrate a fan-beam CT scanner with an angiography suite, address these limitations. Tanaka et al. noted that Angio-CT offers a larger field of view (up to 50 cm) and high-contrast resolution superior to CBCT, mitigating truncated-view artifacts in large patients, albeit at a higher equipment cost and radiation dose (44). Lionberg et al. emphasized that Angio-CT eliminates the need for breath-holding and provides diagnostic-quality multi-phasic imaging, enabling the detection of occult satellite lesions and more precise volumetric delineation for dosimetry (45). Emerging quantitative applications of Angio-CT are also being explored. Campbell et al. reported that the tumor enhancement ratio measured on Angio-CT showed a moderate correlation with the final ^90^Y microsphere distribution observed on positron emission tomography (PET) (r = 0.34), which performed comparably to standard ^99m^Tc-MAA single-photon emission computed tomography (SPECT) -based prediction (r = 0.32) (46). These findings suggest that angio-CT may serve as a quantitative adjunct to conventional imaging for dosimetric assessment and prediction of microsphere distribution, thereby supporting more individualized treatment planning.

## 4 Nuclear medicine imaging for dosimetry and planning in ^90^Y-SIRT

**Figure 2:**
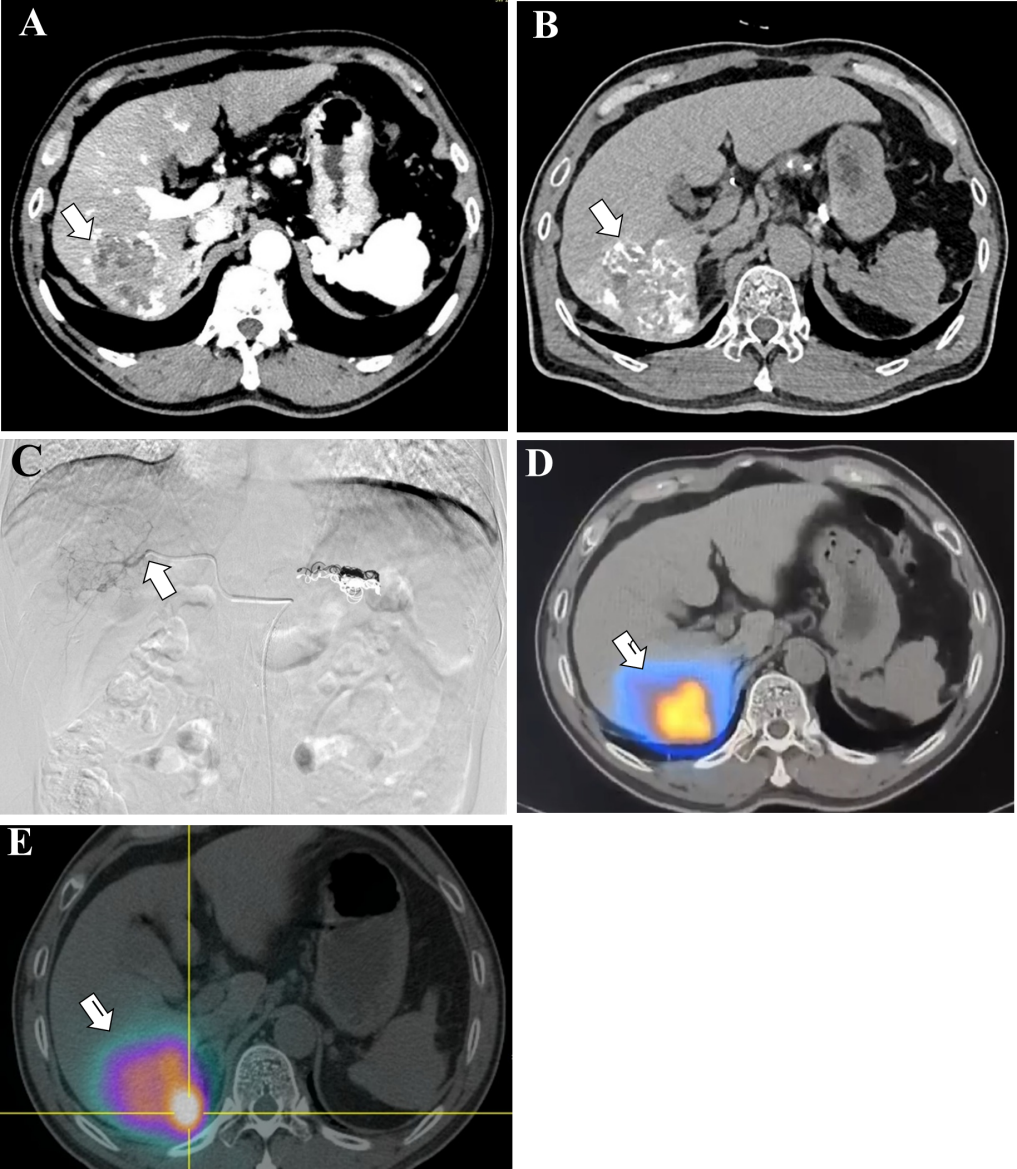
Post-procedural dose verification. Images in a 56-year-old man with hepatocellular carcinoma demonstrate that the intratumoral distribution of ^90^Y microspheres was essentially consistent with the pretreatment ^99m^Tc-MAA prediction. **(A)** Axial contrast-enhanced CT image (arterial phase) shows an arterially enhancing lesion (arrow) in the right hepatic lobe, corresponding to hepatocellular carcinoma. **(B)** Axial Angio-CT image obtained after superselective catheterization of the posterior branch of the right hepatic artery again shows the arterially enhancing lesion (arrow). **(C)** Selective angiogram of the posterior branch of the right hepatic artery shows appropriate catheter position (arrow) for ^99m^Tc-MAA delivery. **(D)** Coregistered ^99m^Tc-MAA SPECT/CT and CT image shows essentially complete tumoral distribution of the ^99m^Tc-MAA (arrow). **(E)** Coregistered posttherapy ^90^Y bremsstrahlung and pretherapy CT image similarly demonstrates similar intratumoral distribution of ^90^Y microspheres (arrow).

Nuclear medicine imaging represents the functional backbone of ^90^Y-SIRT, shifting clinical focus from purely morphological assessment to metabolic and physiological characterization. This “theranostic” approach comprises two distinct phases: pre-treatment simulation using ^99m^Tc-MAA to predict microsphere distribution, and post-treatment verification imaging to validate actual Yttrium-90 deposition. While SPECT has traditionally served as the workhorse for dosimetry and treatment planning, the advent of SPECT/CT hybrid imaging and PET/CT has substantially improved accuracy and enabled more personalized therapy (**Figure 2**).

### 4.1 SPECT/CT imaging

SPECT/CT systems integrate functional data with detailed anatomical localization, overcoming the limitations of planar scintigraphy, such as poor spatial resolution and the lack of tissue attenuation correction. In the context of HCC, where tumor vascularity is complex and often distorted by underlying cirrhosis, this multimodal integration is critical for precise vascular mapping and quantitative dosimetry.

#### 4.1.1 ^99m^Tc- MAA SPECT/CT

^99m^Tc-MAA scintigraphy serves as the mandatory surrogate for simulating microsphere distribution, assessing arterial perfusion, and detecting extrahepatic shunting prior to ^90^Y-SIRT. While planar imaging was historically used, SPECT/CT has demonstrated superior diagnostic performance. Ahmadzadehfar et al. reported that SPECT/CT significantly improved the sensitivity for identifying extrahepatic shunting to 100%, compared to 41% for SPECT and 32% for planar imaging alone (47). Crucially, these findings altered the therapeutic management in 29% of patients, involving coil embolization of aberrant vessels or catheter repositioning, whereas planar imaging only impacted 7.8% of cases (47).

For the detection of gastrointestinal shunting, which poses a risk of severe ulceration, Kao et al. demonstrated that SPECT/CT achieved a sensitivity of 100% and an accuracy of 96%, far exceeding the 25% sensitivity of planar imaging (48). Lenoir et al. further detailed that SPECT/CT effectively identifies non-target uptake in specific anatomical sites, including the gallbladder (12.2%) and the hepatic artery (6.5%) (49). Notably, SPECT/CT is vital for characterizing HCC with portal vein thrombosis; tracer accumulation within the portal vein thrombosis was identified in 6.5% of scans, confirming arterial supply to the thrombus and eligibility for therapy (49).

**Figure 3:**
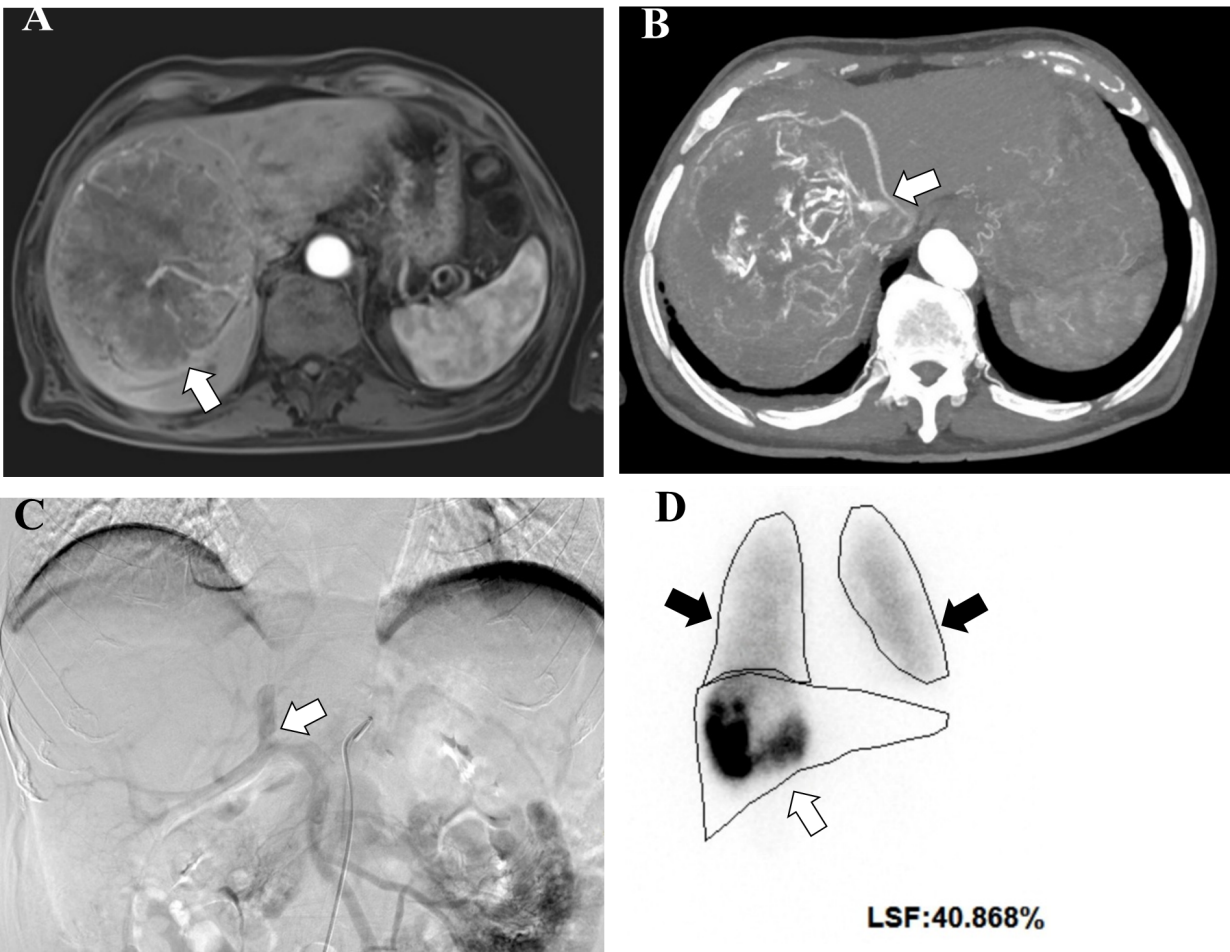
Importance of performing a pre-procedural ^99m^Tc-MAA shunt scan. A 76-year-old man presented with a large hepatocellular carcinoma involving the middle hepatic vein, without extrahepatic disease. **(A)** Axial contrast-enhanced T1-weighted MR image (arterial phase) shows an arterially enhancing lesion (arrow) in the right hepatic lobe, corresponding to hepatocellular carcinoma. **(B)** Axial Angio-CT image (arterial phase) demonstrates early opacification of the main portal vein (arrow), indicating the presence of an arterioportal shunt. **(C)** Digital subtraction angiography performed prior to ^99m^Tc-MAA administration shows the catheter positioned in the superior mesenteric artery. Early opacification of the main portal vein (arrow) is again seen during the arterial phase due to a high-flow shunt during contrast injection. **(D)**^99m^Tc-MAA maximum intensity projection image shows radiotracer activity distributed throughout the right hepatic lobe (white arrow) and in both lungs (black arrows), reflecting extensive portal shunting. The lung shunt fraction was 40.868%, exceeding the safety threshold for ^90^Y radioembolization, and therefore treatment was cancelled.

Quantitative ^99m^Tc-MAA SPECT/CT also plays a pivotal role in personalized dosimetry. Garin et al. established that a tumor-absorbed dose threshold of 205 Gy is highly predictive of treatment response. Patients receiving doses ≥ 205 Gy achieved a significantly prolonged median overall survival of 18 months, compared to 9 months in those receiving lower doses (50). Regarding safety, a lung shunt fraction exceeding 20% typically precludes treatment to prevent radiation pneumonitis (**Figure 3**). However, accurate lung dosimetry allows for treatment in selected cases if the estimated lung dose remains below 30 Gy per session and 50 Gy cumulatively (51). Despite its utility, potential pitfalls such as coregistration errors, observed in 2.1% of cases, require careful image interpretation to avoid false-positive diagnoses (49).

#### 4.1.2 Post-treatment ^90^Y SPECT/CT

Post-treatment imaging of ^90^Y relies on the detection of bremsstrahlung radiation produced by the interaction of β-particles with tissue, as ^90^Y lacks discrete gamma emissions for conventional scintigraphy. While this modality serves as the standard for verifying microsphere deposition, it is technically challenging due to the continuous and broad energy spectrum of bremsstrahlung photons, which leads to significant scatter, septal penetration, and low spatial resolution (52). To mitigate these limitations, Uliel et al. recommend the use of medium-energy collimators and a narrowed energy window to improve image quality and prevent the overestimation of lung shunt fractions caused by scatter (52).

The primary clinical utility of post-treatment ^90^Y SPECT/CT lies in its ability to correct false-positive findings observed on pre-treatment MAA scans, thereby confirming the safety of the procedure. Lenoir et al. demonstrated that while ^99m^Tc-MAA SPECT/CT identified gallbladder uptake in 17 cases and hepatic artery uptake in 9 cases, post-treatment ^90^Y bremsstrahlung imaging confirmed actual microsphere deposition in only 1 case for each site (49). This discrepancy highlights that MAA often overestimates the risk of non-target embolization due to vascular spasms or catheter positioning differences, whereas post-treatment SPECT reflects the true biodistribution (49).

**Figure 4:**
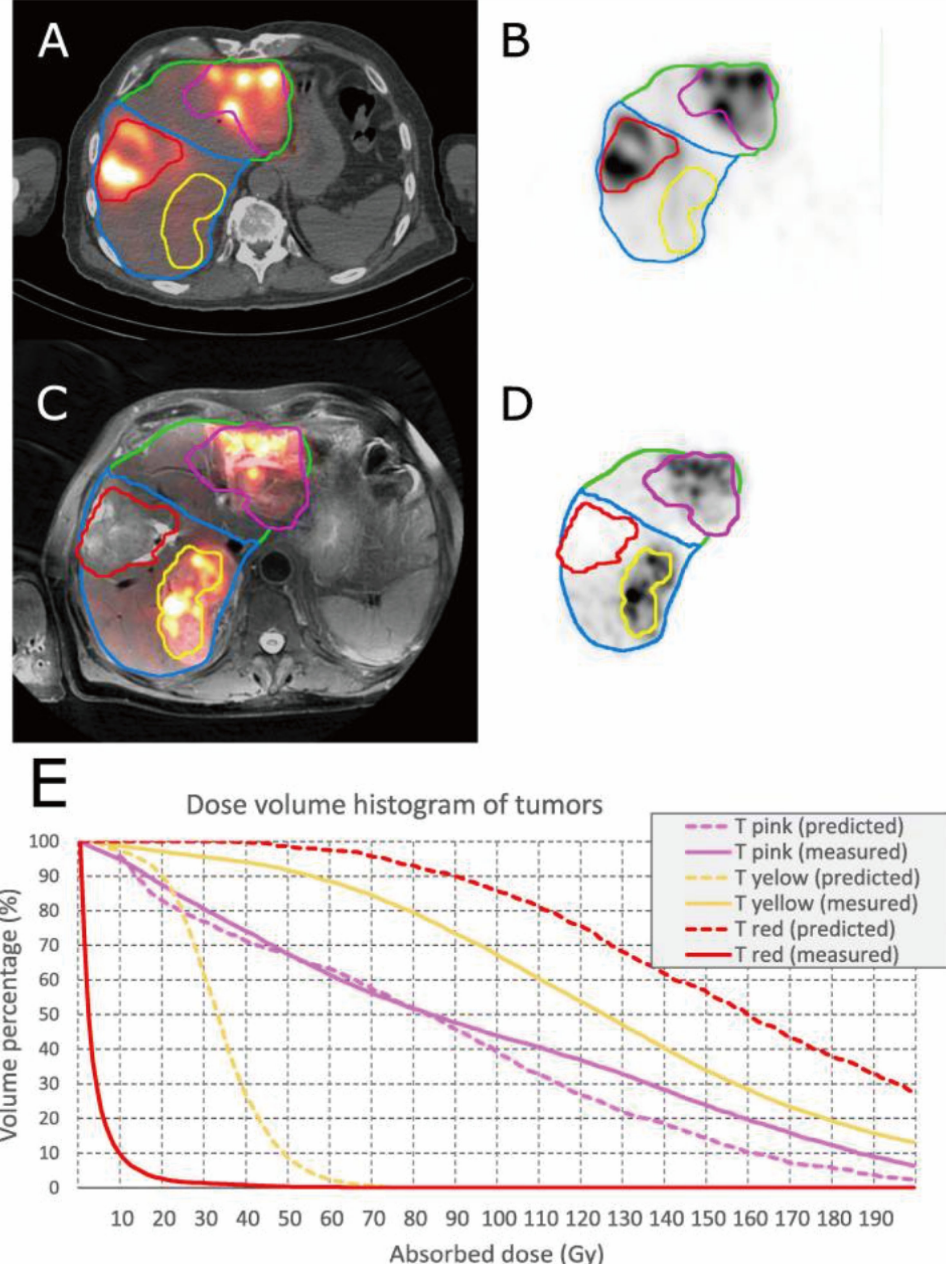
An example of disagreement between tumor dose between predictive and post-treatment dosimetry. **(A, B)** Pre-treatment image (99mTc-MAA-SPECT/CT). **(C, D)** Post-treatment image (^90^Y-PET/MR); the tumor contoured in pink: predictive and post-treatment dose assessment were comparable (pre-treatment predicted: mean dose: 84 Gy, D50 = 83 Gy and V100 = 39%; post-treatment: mean dose: 95 Gy, D50 = 84 Gy and V100 = 44%); the tumor contoured in yellow: predictive dosimetry underestimated the tumor dose (pre-treatment predicted: mean dose: 33 Gy, D50 = 33 Gy and V100 = 0%; post-treatment measurement: mean dose: 130 Gy, D50 = 125 Gy and V100 = 67%); and the tumor contoured in red: predictive dosimetry over-estimated the tumor dose (pre-treatment predicted: mean dose: 165 Gy, D50 = 159 Gy and V100 = 86%; post-treatment: mean dose: 4 Gy, D50 = 2 Gy and V100 = 0%). **(E)** Dose-volume histogram of the described VOIs based on predictive and post-treatment dose assessments. In this case a different catheter positioning between MAA work-up and treatment resulted in flow variation. A preferential targeting of the tumor in the ventral part of the right liver lobe (red tumor) was observed in the work-up. On the other hand, after ^90^Y-microsphere injection, a preferential targeting of the tumor in the dorsal part of the left liver lobe was obtained. This is one example of a potential pitfall in SIRT in general and a cause of discrepancy between ^99m^Tc-MAA and ^90^Y-PET in particular (53). Reproduced from Jafargholi Rangraz E et al., EJNMMI Physics, 2020, CC BY 4.0.

Regarding dosimetry, systematic deviations exist between the predictive ^99m^Tc-MAA model and the actual ^90^Y distribution (53) (**Figure 4**). Wondergem et al. analyzed 39 procedures and found that 68% of liver segments showed a difference greater than 10% between activity predicted by ^99m^Tc-MAA and actual ^90^Y activity, with catheter tip positioning identified as a significant factor influencing this disagreement (54). Similarly, Debebe et al. reported that the mean tumor-to-normal liver ratio (TNR) derived from ^99m^Tc-MAA (9.4) was significantly higher than that measured on post-treatment ^90^Y SPECT (5.0), suggesting that ^99m^Tc-MAA tends to overestimate tumor uptake (55). This tendency was corroborated by recent studies. Cheng et al. found that pre-treatment ^99m^Tc-MAA SPECT/CT yielded a higher median TNR (5.8) compared to post-treatment verification imaging (4.9), although it accurately predicted the absorbed dose to normal liver tissue (56). Similarly, Demir et al. observed that while ^99m^Tc-MAA SPECT/CT provided reliable dose estimates for healthy liver parenchyma, it showed lower accuracy for tumor-absorbed doses, with D90 values calculated via SPECT being systematically higher than those from PET-based verification (57). In terms of volumetric dosimetry, achieving the minimum dose to 70% of the tumor volume (D70) of 95 Gy has been associated with objective response, whereas non-responders had a significantly lower mean D70 of 60 Gy (58). However, due to the inherent quantitative limitations of bremsstrahlung imaging, Elschot et al. noted that SPECT underestimates the dose in small lesions by up to 75%, necessitating the adoption of more advanced modalities like PET/CT for precise voxel-based dosimetry (59).

### 4.2 PET/CT imaging

#### 4.2.1 Tumor biology and risk stratification

While conventional anatomical imaging guides catheter navigation, PET/CT provides critical insights into tumor metabolic activity and biological aggressiveness, serving as a powerful tool for patient selection and risk stratification. Although ^18^F-fluorodeoxyglucose (^18^F-FDG) PET has limited sensitivity (50-65%) for detecting well-differentiated HCC due to high glucose-6-phosphatase activity, Filippi et al. emphasize its value in identifying aggressive, poorly differentiated phenotypes that are associated with microvascular invasion and poor outcomes (60).

High FDG uptake is a robust independent predictor of survival, often outperforming traditional staging systems. In a multicenter cohort of 291 patients with BCLC stage C HCC, Na et al. demonstrated that patients with a low tumor-to-liver uptake ratio (TLR < 3.0) had a median overall survival of 14.9 months, significantly longer than the 6.4 months observed in patients with high TLR (61). In the specific context of ^90^Y-SIRT, Jreige et al. reported that metabolic parameters are superior to morphological criteria for predicting long-term survival. In their study, patients with low maximum standardized uptake value achieved a median overall survival of 30.9 months, compared to only 9 months for those with high metabolic activity (P = 0.02) (62). These findings suggest that patients with high FDG avidity represent a high-risk subgroup that may require treatment intensification or combination therapy.

For well-differentiated tumors that are often FDG-negative, ¹¹C-acetate or ^18^F-fluorocholine (^18^;F-FCH) serve as effective surrogates for cell membrane biosynthesis, offering higher sensitivity for detection (60). Furthermore, PET/CT is highly effective for early risk stratification post-treatment. Reizine et al. showed that early evaluation (4–8 weeks post-SIRT) using ^18^F-FDG or ^18^F-FCH PET/CT could predict 6-month response with 100% sensitivity and 100% specificity, whereas standard mRECIST assessment at 1 month showed lower diagnostic accuracy (63).

#### 4.2.2 Post-treatment ^90^Y PET/CT

Although Yttrium-90 is a pure beta-emitter, it undergoes a minor decay branch (approximately 32 per million decays) generating internal pair production, which allows for direct imaging of microsphere biodistribution using PET scanners (64). This modality overcomes the fundamental resolution limitations of bremsstrahlung SPECT. Elschot et al. quantitatively compared the two modalities in a phantom study, demonstrating that while SPECT underestimated the absorbed dose in small spheres (10 mm) by 75%, Time-of-flight (TOF) PET reduced this underestimation to 45%, while achieving an error rate of only 11%in larger spheres (37 mm) (59). Furthermore, the multicenter QUEST phantom study confirmed that current-generation TOF-PET scanners can consistently reconstruct ^90^Y activity concentrations; however, activity concentrations in small structures (≤ 37 mm) are systematically underestimated, by approximately 20% for spheres larger than 20 mm, due to partial volume effects (65).

The superior spatial resolution of ^90^Y PET/CT facilitates precise voxel-based dosimetry and the generation of dose-volume histograms (DVH), which are critical for predicting treatment efficacy. Kao et al. analyzed post-treatment PET dosimetry in HCC patients and identified a clear dose-response relationship: complete tumor responses were generally achieved when D70 exceeded 100 Gy, while non-responders typically had a D70 below 100 Gy (64). This study also established safety thresholds, noting that a mean dose of 49 Gy to the stomach was associated with gastritis, while 18 Gy was asymptomatic (64). A recent systematic review by Roosen et al. further corroborated these findings, suggesting that a target mean dose of 100–250 Gy is generally required to induce a response in HCC, a threshold significantly higher than that for colorectal liver metastases (40–60 Gy) (66). Additionally, post-treatment PET dosimetry correlates well with metabolic response; Levillain et al. reported a strong correlation (R² = 0.82) between the mean absorbed dose and the reduction in total lesion glycolysis, identifying 39 Gy and 60 Gy as key metabolic response thresholds (67).

Recent technological advancements, such as long axial field-of-view (LAFOV) PET/CT systems, further enhance the utility of post-treatment imaging by offering higher sensitivity and total-body coverage. Linder et al. demonstrated that LAFOV systems allow for a reduced scan duration of 20 minutes while maintaining adequate image quality and quantification accuracy for dosimetry (68). However, challenges remain in voxel-wise dosimetry algorithms. In a 2025 comparative study, Zeimpekis et al. reported that despite the high sensitivity, current voxel-wise algorithms on LAFOV systems may significantly underestimate the delivered dose compared to pre-treatment SPECT planning. Specifically, the median tumor D90 measured by LAFOV PET was significantly lower than that predicted by SPECT (30.5 Gy vs 123.5 Gy), suggesting that further refinement of dosimetric tools is necessary to fully leverage this advanced technology for accurate absolute dose quantification (69).

## 5 Multimodal imaging integration across the ^90^Y-SIRT workflow

The precision of ^90^Y-SIRT relies on the accurate definition of the biological target volume during the pre-procedural phase, a task where the integration of anatomical and functional imaging significantly outperforms single-modality assessment (11). While CT serves as the standard for vascular mapping, it often fails to capture the full extent of viable tumor burden in cirrhotic livers. Basha et al. demonstrated that a combined CT and MRI protocol yields a diagnostic accuracy of 91.29%, significantly superior to the 67.6% accuracy of CT alone (70). This multimodal approach is particularly critical for characterizing “borderline” hepatic nodules; Joo et al. revealed that 44.0% of hepatobiliary phase hypointense nodules without arterial enhancement—often dismissed as benign on standard CT—are pathologically confirmed as progressed HCC (71). Consequently, integrating hepatobiliary phase MRI with ^99m^Tc-MAA SPECT/CT is essential for personalized dosimetry. Although ^99m^Tc-MAA SPECT/CT generally correlates well with final ^90^Y biodistribution, Cheng et al. cautioned that it tends to overestimate the tumor-to-normal ratio in the left hepatic lobe, necessitating the cross-referencing of functional SPECT data with high-resolution anatomical MRI to prevent non-target radiation (56).

**Figure 5:**
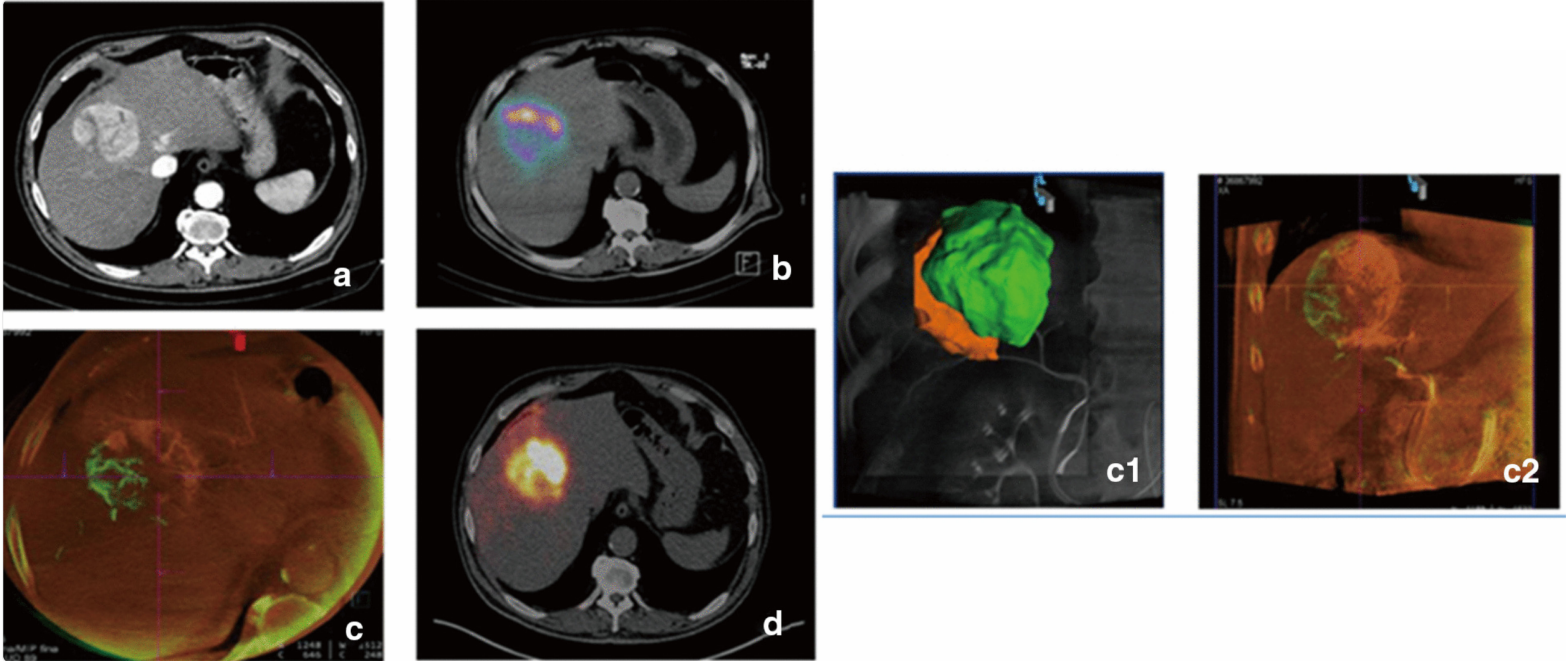
**(a)** Contrast-enhanced computed tomography (CECT) image: HCC located between segments IV and VIII. **(b)**^99m^Tc-MAA SPECT/CT fusion image shows low uptake in the lateral part of the tumoral nodule. ^99m^Tc-MAA activity was split in two doses of 50% each by IR decision, based on liver and tumoral volumes. (c, c1 and c2) C-arm cone-Beam CT (CBCT) volumetric assessment of the tumoral territory perfused by each artery. VIII segments artery (in green) fed only 32% of the tumoral volume while IV segment artery fed most of it (in orange). **(d)**^90^Y PET/CT fusion image after splitting the activity according to CBCT volumes shows the adequate distribution of the microspheres throughout the lesion (80). Reproduced from Rodríguez−Fraile et al., EJNMMI Research, 2021, CC BY 4.0.

Translating these complex pre-operative plans into precise catheter delivery requires real-time intra-procedural navigation, where volumetric imaging acts as the vital link between diagnostic planning and angiographic execution. The application of “fusion guidance”, which overlays pre-operative cross-sectional volumes onto live fluoroscopy, transforms 2D angiography into a 3D navigational environment. Within this workflow, the joint application of functional and anatomical modalities is decisive. Rodríguez-Fraile et al. highlighted that integrating intra-procedural CBCT with pre-treatment ^99m^Tc-MAA SPECT/CT optimizes dosimetric planning; specifically, the anatomical detail from CBCT compelled a modification of the treatment plan—such as the splitting of administered activity—in 42% of patients with multiple feeding arteries, ensuring complete tumor coverage that would have been ambiguous on SPECT alone (72) (**Figure 5**). Furthermore, fusing unenhanced CBCT with pre-treatment contrast-enhanced CT enhances the sensitivity for detecting small viable tumor foci to 96.5%, significantly superior to the 78.6% sensitivity of standard MDCT (73), thereby minimizing the risk of geographic miss during the intervention.

Following the procedure, the focus shifts to quantitative verification, where the superior spatial resolution of PET-based modalities allows for voxel-level dosimetric validation that far exceeds the capabilities of traditional Bremsstrahlung SPECT. ^90^Y PET/CT has established a clear dose-response relationship, with Kokabi et al. identifying a mean absorbed tumor dose of 253 Gy as a robust threshold for predicting objective response (AUC = 0.929) (74). Moving beyond average doses, the emergence of simultaneous PET/MRI offers a deeper insight into dose heterogeneity. Demir et al. reported that while pre-treatment SPECT accurately predicts the dose to normal liver parenchyma, it fails to capture the non-uniformity of microsphere deposition within the tumor; post-treatment ^90^Y PET/MRI detected significantly higher “hot spot” doses compared to SPECT/CT (57). This capability to generate accurate DVH allows clinicians to distinguish between true dosimetric failure and heterogeneous coverage, as validated by Fowler et al. in predicting individual lesion response (75).

Finally, functional imaging provides a critical advantage in the early post-treatment window by detecting physiological responses that precede morphological tumor shrinkage. Traditional size-based criteria such as RECIST are often insensitive in the first few months due to treatment-induced edema and necrosis. In contrast, functional changes serve as immediate biomarkers of efficacy. Rhee et al. demonstrated that DWI can predict response as early as 1-month post-treatment, where a 10.5% increase in ADC values identified responders with 93% sensitivity (76). Similarly, CEUS offers an accessible bedside alternative; Delaney et al. showed that CEUS can detect a significant reduction in fractional vascularity (approximately 38%) in responders as early as 2 weeks after therapy (16). These functional metrics enable the early identification of non-responders, facilitating timely adjunctive therapies rather than waiting for delayed anatomical progression.

## 6 Conclusions and future perspectives

The evolution of Yttrium-90 radioembolization for hepatocellular carcinoma marks a paradigm shift where therapeutic efficacy increasingly depends on the depth and integration of imaging data. The transition from empirical treatment to precision oncology relies on a multimodal imaging framework transcending simple anatomical visualization. While conventional modalities like angiography and CT provide essential vascular roadmaps, they offer incomplete insights into tumor biology and metabolic activity when used in isolation. The synergistic application of functional imaging (DWI, CEUS) and molecular imaging (SPECT/CT, PET/CT) is therefore critical for constructing a comprehensive “theranostic” profile. This integration facilitates accurate definition of the biological target volume, reducing risks of geographic miss and non-target embolization.

Multimodal imaging is perhaps most valuable in mitigating inherent procedural uncertainties. Synthesizing pre-procedural functional data with intra-procedural volumetric imaging creates a validation loop, ensuring the treatment plan aligns with the actual vascular territory. The growing adoption of post-treatment quantitative validation, particularly voxel-based ^90^Y-PET/CT dosimetry, provides a robust mechanism to correlate absorbed dose with pathological response. This shift towards quantitative dosimetry transforms imaging from a passive observation tool into an active biomarker of therapeutic success, distinguishing technical limitations from biological non-response.

Looking forward, the maturation of ^90^Y-SIRT will hinge on standardizing quantitative imaging protocols across institutions to ensure reproducibility (77). Technical advancements, such as simultaneous PET/MRI systems, offer a promising avenue to further refine dosimetric accuracy by minimizing motion-related registration errors and improving soft-tissue contrast (75). Additionally, the emerging field of radiomics and artificial intelligence represents a potential supplementary tool for treatment optimization. Rather than replacing conventional interpretation, these computational analyses may help uncover subtle, non-invasive imaging phenotypes capable of predicting tumor aggressiveness or radio-resistance (78). The future of ^90^Y-SIRT lies in the continued refinement of this multimodal ecosystem, where imaging data drives a personalized, predictable, and effective therapeutic strategy for patients with hepatocellular carcinoma.
